# Differences Among Health Care Settings in Utilization and Type of Physical Rehabilitation Administered to Patients Receiving Workers’ Compensation for Musculoskeletal Disorders

**DOI:** 10.1007/s10926-012-9412-y

**Published:** 2013-01-18

**Authors:** Paul F. Beattie, Roger M. Nelson, Kevin Basile

**Affiliations:** 1 Doctoral Program in Physical Therapy, Arnold School of Public Health, University of South Carolina, Columbia, SC 29208 USA; 2 Clinical Benchmarks, LLC, King of Prussia, PA 19406 USA; 3 Lebanon Valley College, Annville, PA 17003 USA; 4 MedRisk, Inc., King of Prussia, PA 19406 USA

**Keywords:** Musculoskeletal injury, Injured worker, Health services, Rehabilitation

## Abstract

*Introduction* There is a paucity of data describing the relationship between practice setting and the delivery of physical rehabilitation to injured workers. *Purpose* To determine differences in the number of visits, the number of treatment units, and the proportion of billing for physical agents over an episode of care between different practice settings’ providing physical rehabilitation to patients receiving workers’ compensation for a musculoskeletal problem. *Methods* A large administrative database was evaluated retrospectively. Practice settings were classified as physician office, corporate physical therapy clinic, occupational medicine clinic, hospital-based outpatient clinic, or private physical therapy practice. *Results* 70,306 subjects (72.7 % male; mean age = 44.6, SD = 11.8 years) were included in this study. Corporate physical therapy clinics had the highest mean values for total visits (13.1, SD = 12.7) and for total units (66.8, SD = 85.5), and the lowest mean values for proportion of physical agents during the episode of care (.22, SD = .18). Occupational medicine clinics had the lowest mean values for total visits (6.8, SD = 7.9) and for total units (30.4, SD = 36.5), and the highest mean value for proportion of physical agents during the episode of care (.41, SD = .22). When controlling for ICD-9-CM codes, body-part treated, surgical status, and geographical region there were small changes in effect size; however, the significance and directionality of differences between practice settings were not changed. *Conclusions* There were significant differences in billing for physical rehabilitation services between practice settings for patients receiving workers’ compensation. Corporate physical therapy clinics billed for more total visits and total units over an episode of care than did other practice settings; however they also billed for a lower proportion of physical agents indicating a greater use of those interventions supported by evidence-based guidelines (exercise and manual therapy) compared to other practice settings.

## Introduction

Musculoskeletal injuries are a primary reason for individuals to receive medical care covered by workers’ compensation [1–4]. Physical rehabilitation is a frequent component of the non-operative, and post-surgical management of many people with these conditions [5–7]. Although the efficacy and effectiveness of many physical rehabilitation interventions for musculoskeletal disorders have been described, there is considerable debate regarding the optimal clinical setting in which these interventions should be delivered to injured workers [8–17]. For example, in the United States, workers’ compensation can be billed for physical rehabilitation that is performed in a wide variety of settings including, but not limited to, a private physical therapy practice, a corporate physical therapy clinic, a hospital-based clinic, a physician’s office or an occupational medicine clinic. It has been frequently argued that variations in the business model of these clinical settings may impact on the overall cost-value of physical rehabilitation care provided [8, 10, 11, 14, 15]. Proponents of this argument submit that certain clinical settings are more likely to “over-utilize treatments” by having the patient receive an excessive number of visits and treatment units [11, 14]. In addition, it has been argued, but not demonstrated, that certain clinical settings over-rely upon time-effective but poorly supported treatments such as “physical agents,” i.e., electrotherapy, thermotherapy and hydrotherapy, rather than evidence-supported, but more time-intensive treatments such as exercise and manual therapy [14].

Despite the potentially important impact upon clinical practice guidelines and health-care policy, there is a paucity of published data addressing the role of the clinical setting in the utilization of physical rehabilitation and in the adherence to evidence-based guidelines for specific types of interventions used [18–20]. Clarification of the relationship of the clinical setting to the delivery of physical rehabilitation for injured workers would fill an important research gap and greatly assist in the development of conceptual models that would help to maximize the cost-value of care. The purpose of the current study is to determine differences in the number of visits, number of treatment units, and proportion of billing for physical agents over an episode of care between different practice settings’ providing physical rehabilitation to patients receiving workers’ compensation for a musculoskeletal problem.

## Methods

### Study Design and Database Construction

A retrospective, cross-sectional evaluation was performed using bill payment records de-identified to protect patient privacy. Subjects were included if they were at least 18 years of age, and had completed an episode of physical rehabilitation for a musculoskeletal problem covered by workers’ compensation. The dataset was generated from the bill-pay activity resulting from 11 nationally based insurance carriers that cover workers’ compensation, and represented claims from 49 of the 50 United States (Rhode Island has a different coding system than others and was not included) and the District of Columbia submitted between July 2009 and December 2011. All cases represented a single episode of care at only one facility for the present claim. Cases with more than one episode of care for their claim, or those who received care at more than one facility for their current claim were not included in the analysis. Billing was based upon dates of service; bundled billing was not used in this system.

### Measures

#### Dependent Variables

Physical rehabilitation utilization was measured by the number of visits and the total number of units billed for during the episode of care. Physical rehabilitation treatments were identified using the Current Procedural Terminology (CPT) codes included within the 97000 series [21] (Table 1). The treatments were then classified into one of two categories. The first category was labeled “physical agents” and included treatments employing heat, light, sound or electricity. Common examples of physical agents include hot packs, laser, ultrasound and transcutaneous electrical stimulation (TENS) [22]. The second category was labeled “therapeutic procedures” and included treatments utilizing exercise or manual therapy (joint mobilization, manipulation or massage). The total units billed for physical agents during the episode of care were divided by the total treatment units billed for during the episode of care to provide a representation of the proportion of total treatment that was devoted to physical agents. The proportion of treatment not devoted to physical agents represented treatment with therapeutic procedures. For example, if the proportion of physical agents to total units was .30, this would indicate that 30 % of the units billed during the episode of care where for physical agents and the remaining 70 % were billed for therapeutic procedures.

**Table 1:** Physical therapy treatment procedures and their corresponding 97000 code classified as “physical agents” or “therapeutic procedures”

Physical agents	97000 code	Therapeutic procedures	97000 code
Hot and cold packs	97010	Therapeutic procedure	97110
Electrical stimulation (unattended)	97014	Neuromuscular reeducation	97112
Paraffin bath	97018	Aquatic therapy	97113
Whirlpool	97022	Gait training	97116
Diathermy	97024	Massage	97124
Infrared	97026	Manual therapy	97140
Ultraviolet	97028	Therapeutic procedure (group)	97150
Electrical stimulation (attended)	97032	Therapeutic activity	97530
Iontophoresis	97033	Development of cognitive skill	97532
Contrast bath	97034	Sensory integrative techniques	97533
Ultrasound	97035	Self care/home management	97535
Mechanical traction	97012	Community/work reintegration	97537
Laser (unlisted modality)	97039		

#### Independent Variables

Physical rehabilitation practice settings were classified based upon billing records. Each claim was classified into one of the following: physician office, corporate physical therapy clinic, occupational medicine clinic, hospital-based outpatient clinic, or private physical therapy practice.

The primary diagnosis for which care was provided was identified by the International Classification of Disease, 9th revision [23], Clinical Modification (ICD-9-CM) code that was submitted by the provider during the initial evaluation by physical rehabilitation services. Because the large number of ICD-9-CM codes within the musculoskeletal domain resulted in a low number of responses for many categories we chose to collapse our classification to increase statistical power and interpretability. The collapsed classification included 5 categories that are similar to those described by Pendergast et al. [18] and included: arthropathy (arthritis or joint problems), dorsopathy (spine or back disease), sprains or strains, fractures or dislocations, and other.

To provide additional information regarding the nature of subjects’ clinical condition we also classed subjects based upon the location of the body-part (s) for which care was provided. This classification included the upper extremity, lower extremity, back, neck, hand or multiple body-parts. Subjects were further classified based upon the presence or absence of a surgical procedure associated with their claim as well as the geographic region of the United States in which they received care.

### Statistical Analysis

The initial dataset was evaluated and those cases with missing values or primary ICD-9-CM codes that were not within the musculoskeletal domain were deleted. Remaining data were summarized and the distributions of dependent variables were checked for normality. Logarithmic transformations were made to non-normal distributions.

Differences in the distributions or frequencies of variables describing subject characteristics (age-groups, gender, chronicity (days from onset of symptoms to beginning of rehabilitation treatment), surgical treatment or not, history of a prior W/C claim, and ICD-9-CM classification) between practice settings were investigated using one-way analysis of variance (ANOVA) or Chi square analysis.

Unadjusted differences in the distributions of each of the dependent measures (total visits, total units, and units of physical agents/total units per episode) between practice settings were assessed using one-way ANOVAs with Schieffe post hoc analysis. Univariate, general linear model two-way ANOVAs were then used to evaluate the between-group differences for practice settings for each of the dependent measures, i.e. total visits, total units, and proportion of total treatment units devoted to physical agents when adjusting for diagnosis, the body-part that was treated, surgical status and geographic region in which care was provided. The alpha level for all comparisons was set at .01. All analyses were performed using IBM SPSS version 19.0.

## Results

### Sample Characteristics

Claims from a total of 3,944 clinical facilities were included. The majority of clinical facilities were from private physical therapy practices (n = 2,860, 72.5 %), followed by corporate physical therapy clinics (n = 561, 14.2 %), physician offices (n = 263, 6.7 %), hospital-based outpatient clinics (n = 180, 4.6 %), and occupational medicine clinics (n = 80, 2.0 %). The initial data set consisted of 76,667 subjects. Of that, 6,361 claims were deleted due to missing values or primary ICD-9-CM codes that were not within the musculoskeletal domain. That elimination resulted in a dataset of 70,306 subjects used for analysis. Of the 49 U.S. states and the District of Columbia that were represented in this sample, 50.1 % were from California, Florida, New Jersey or Pennsylvania.

Males comprised 72.7 % of the subjects (n = 51,332). The mean age of all subjects was 44.6 years (SD = 11.8). Males were slightly older than females (mean = 44.9, SD = 11.6 years vs. mean = 44.0, SD = 12.2 years; *p* < .01). The highest percentage of subjects received care from private physical therapy practices (53.7 % of the total sample), followed by corporate physical therapy clinics 30.7 %, physician offices 7.3 %, occupational medicine clinics 5.0 %, and hospital-based clinics 3.3 %. A total of 42.9 % of subjects (n = 30,143) began physical therapy more than 90 days after symptom onset. The most frequent body-part (s) treated was “multiple” (41.4 %, n = 29,083) followed by “back” (29.0 %, n = 20,418). 23.3 % of patients (n = 16,379) received physical therapy following surgical intervention, and 19.2 % (n = 13,528) had a history of a prior workers’ compensation claim (Table 2).

**Table 2:** Subject characteristics for each of the practice settings

Characteristic	Physician offices	Corporate physical therapy clinics	Occupational medicine	Hospital-based	Private physical therapy practices	Total	
Gender	Male	3,621	15,744	2,449	1,738*	27,616*	51,168
	Female	1,478*	5,875	1,079*	587	10,119	19,138
Age group (years)	18–30	725	3,104	696*	404*	5,297*	10,226
	31–40	1,022	4,551	878*	473	8,072*	14,996
	41–50	1,541	6,671*	1,029	674	11,488	21,403
	51–60	1,300*	5,410*	685	583*	9,448*	17,426
	Over 60	511*	1,883	240	191	3,430*	6,255
Degree of chronicity (days)	Acute 0–30	1,508	7,191*	2,405*	966*	10,274	22,344
	Sub-acute 31–90	1,130	5,561*	555	573	10,000*	17,819
	Chronic >90	2,461*	8,867	568	786	17,461*	30,143
Body segment treated	UE	673*	2,754	422	314*	5,023*	9,186
	LE	578*	2,234*	430*	222	3,456	6,920
	Back	1,643*	6,100	1,284*	739*	10,652	20,418
	Neck	241*	740	58	45	1,330*	2,414
	Hand	188*	712*	289*	95*	1,001	2,285
	Multiple	1,776	9,079*	1,045	910	16,273*	29,083
Surgical intervention	Yes	1021	5,269*	142	386	9,561*	16,379
	No	4,078*	16,350	3,386*	1,939*	28,174	53,927
Prior W/C Claim	No	4,121*	17,084	2,756	1,891*	30,926*	56,778
	Yes	978	4,535*	772	434*	6,809	13,528

Cells with observed frequency exceeding expected frequency are highlighted by * (*p* < .01)

38.25 % (n = 26,940) of subjects’ conditions were classified as sprains or strains. 31.3 % (n = 22,090) were classified as arthropathy, 21.1 % (n = 14,855) were classified as dorsopathy, and the remainder were classified as fractures or dislocations (9.0 % n = 6,322) or other (0.5 %, n = 360) (Table 3). Arthropathy was most frequently observed in those patients who received treatment to multiple body-parts, while dorsopathy most frequently observed in those patients receiving care to the back or neck. Sprains and strains were most frequently observed in the back, upper and lower extremities and hand, while fractures and dislocations were most frequently observed in the upper and lower extremities (Table 4).

**Table 3:** The frequency of diagnoses using the collapsed ICD-9-CM codes for each of the practice settings

Diagnosis	Physician offices	Corporate physical therapy clinics	Occupational medicine clinics	Hospital-based clinics	Private physical therapy practices	Total
Arthropathy	1,340	6,941*	566	677	12,464*	21,988
Dorsopathy	926	4,712*	351	381	8,430*	14,800
Sprains and strains	2,434*	7,818	2,479*	1,086*	13,038	26,855
Fractures and dislocations	383	2,049*	106	167	3,599*	6,304
Other	16	99	26*	14*	204*	359
Total	21,619	3,528	2,325	37,735	70,306	

Cells with observed frequency exceeding expected frequency are highlighted by * (*p* < .01)

**Table 4:** The diagnostic classification X the body-part that was treated for the entire sample

Diagnosis classification	Upper extremity	Lower extremity	Back	Neck	Hand	Multiple	Total
Arthropathy	0	319	42	0	0	21,627*	21,988
Dorsopathy	0	0	8,217*	2,414*	0	4,169	14,800
Sprains and strains	4,947*	4,833*	12,159*	0	2,216*	2,700	26,855
Fractures and dislocations	4,194*	1764*	0	0	63	283	6,304
Other	45	4	0	0	6	304*	359
Total	9,186	6,920	20,418	2,414	2,285	29,083	70,306

Cells with observed frequency exceeding expected frequency are highlighted by * (*p* < .01)

### Unadjusted Differences in Billing Between Practice Settings

#### Number of Visits per Episode

Hospital-based clinics and physician offices were not significantly different in the number of visits per episode of care. All other comparisons were different at the *p* < .001 level. Corporate physical therapy clinics had a significantly higher mean number of visits during the episode of care (mean = 13.08, SD = 12.73) than any of the other clinical settings (Table 5). The 95 %-confidence intervals of the differences between corporate physical therapy clinics and other clinical settings ranged from 0.6 to 1.2 fewer visits for private physical therapy practices to 5.7–7.0 fewer visits for occupational medicine clinics.

**Table 5:** The mean (standard deviation) for primary dependent measures over the episode of care for each of the practice settings

Practice setting	Number of cases	Visits per episode	SD	Units per episode	SD	Physical agents/total units for episode	SD
Physician offices	5,099	10.47	11.29	42.73	57.17	.34	.24
Corporate physical therapy clinics	21,618	13.08	12.73	66.79	85.54	.22	.18
Occupational medicine clinics	3,528	6.77	7.93	30.47	36.52	.41	.22
Hospital-based clinics	2,325	10.17	11.13	43.27	67.64	.31	.24
Private physical therapy practices	37,735	12.18	12.12	51.38	62.22	.28	.22
Total	70,305	12.00	12.13	54.17	69.84	.28	.21

#### Number of Treatment Units per Episode

Hospital-based clinics and physician offices were not significantly different in the number visits per episode of care. All other comparisons were different at the *p* < .001 level. Corporate physical therapy clinics had a significantly higher mean of units during the episode of care (mean = 66.79, SD = 85.54) than any of the other clinical settings (Table 5). The 95 %-confidence intervals of the differences between corporate physical therapy clinics and other clinical settings ranged from 13.7 to 17.3 fewer units for private physical therapy practices to 32.4–40.2 fewer units for occupational medicine clinics.

#### Proportion of Units of Physical Agents to Total Units for the Episode of Care

All comparisons were different at the *p* < .001 level. Occupational medicine clinics had the highest mean proportion of units of physical agents to total units (mean = .41, SD = .22) Corporate physical therapy clinics had the lowest mean proportion of physical agents (mean = .22, SD = .18) followed by private physical therapy practices (mean = .28, SD = .22). The 95 %-confidence intervals of the differences between occupational medicine practices and other clinical settings ranged from 0.04 to 0.07 less for physician offices to .17–.19 less for corporate physical therapy clinics (Table 5).

### Differences in Billing Between Practice Setting Adjusted for ICD-9-CM Codes

#### Number of Visits per Episode

Corporate physical therapy clinics had a significantly higher mean number of visits during the episode of care for subjects classified as arthropathy and sprains or strains compared to the other clinical settings (Table 6). The 95 %-confidence intervals of the differences between corporate clinics and other clinical settings for arthropathy ranged from 0.80 to 2.06 fewer visits for private physical therapy practices to 5.67–9.35 fewer visits for occupational medicine clinics. The 95 %-confidence intervals of the differences between corporate clinics and other clinical settings for sprains or strains ranged from 0.17 to 1.05 fewer visits for private physical therapy practices to 3.61–5.02 fewer visits for occupational medicine clinics.

**Table 6:** The mean (standard deviation) for the primary measures by practice setting and ICD-9-CM codes

Practice setting	Diagnosis	Number of cases	Mean visits per episode	SD	Mean units per episode	SD	Ratio passive/total units	SD
Physician offices	Arthropathy	1,340	12.79	13.32	52.92	68.57	.30	.23
	Dorsopathy	926	9.86	9.36	38.31	44.30	.29	.25
	Sprains and strains	2,434	9.00	10.29	37.09	53.32	.40	.24
	Fractures and dislocations	383	13.31	12.22	54.01	59.00	.25	.22
	Other	16	8.19	7.29	32.06	35.30	.40	.21
	Total	5,099	10.47	11.29	42.73	57.17	.34	.24
Corporate physical therapy clinics	Arthropathy	6,941	15.70	14.59	82.20	98.97	.22	.18
	Dorsopathy	4,712	11.90	11.19	58.75	66.87	.21	.18
	Sprains and strains	7,817	10.51	10.25	51.74	64.30	.24	.18
	Fractures and dislocations	2,049	16.72	15.05	90.49	123.93	.17	.15
	Other	99	13.18	11.84	66.49	69.69	.26	.22
	Total	21,618	13.08	12.73	66.79	85.54	.22	.18
Occupational medicine	Arthropathy	566	8.20	11.40	37.04	50.03	.43	.20
	Dorsopathy	351	7.41	8.96	30.17	34.33	.42	.19
	Sprains and strains	2,479	6.19	6.08	28.55	31.28	.40	.22
	Fractures and dislocations	106	10.81	15.32	43.20	61.11	.35	.23
	Other	26	5.50	3.71	22.42	15.96	.37	.19
	Total	3,528	6.77	7.93	30.47	36.52	.40	.22
Hospital-based	Arthropathy	677	12.16	12.19	51.17	64.22	.26	.23
	Dorsopathy	381	10.94	11.49	46.93	87.16	.26	.24
	Sprains and strains	1,086	7.83	8.98	32.84	56.66	.36	.23
	Fractures and dislocations	167	15.71	14.43	70.56	82.97	.19	.19
	Other	14	8.71	8.99	46.00	57.80	.24	.32
	Total	2,325	10.17	11.13	43.27	67.64	.30	.24
Private physical therapy practices	Arthropathy	12,464	14.30	13.35	60.70	70.67	.26	.21
	Dorsopathy	8,430	11.34	11.23	46.76	55.25	.28	.23
	Sprains and strains	13,038	9.90	10.37	41.65	51.94	.31	.22
	Fractures and dislocations	3,599	14.99	13.51	64.52	71.92	.22	.20
	Other	204	14.03	13.93	61.65	75.77	.31	.22
	Total	37,735	12.18	12.11	51.37	62.22	.28	.22
Entire sample	Arthropathy	21,988	14.43	13.74	66.11	80.85	.25	.20
	Dorsopathy	14,800	11.33	11.09	49.66	59.63	.26	.22
	Sprains and strains	26,854	9.57	10.03	42.61	55.14	.31	.22
	Fractures and dislocations	6,304	15.40	14.05	72.12	92.60	.21	.19
	Other	359	12.71	12.68	58.22	70.22	.30	.22
	Total	70,305	12.00	12.13	54.17	69.84	.27	.21

There were no significant differences between corporate physical therapy clinics, hospital-based clinics and private physical therapy practices regarding the number of visits for patients classified as dorsopathy. Each of these settings had higher means than physician offices and occupational medicine clinics. Corporate physical therapy clinics and hospital-based clinics had higher means than the other settings relative to the number of visits for patients treated for fractures or dislocations, but were not different from one another. There were no significant differences in the number of visits among these settings groups for patients classified as other.

#### Number of Treatment Units per Episode

Corporate physical therapy clinics had a significantly higher mean number of units during the episode of care for each of the ICD-9 classifications except fractures or dislocations, and other, when compared with the remaining clinical settings (Table 6). The 95 %-confidence intervals of the difference between corporate physical therapy clinics and other clinical settings for arthropathy ranged from 17.9 to 25.3 fewer units for private physical therapy practices to 34.4–55.9 fewer units for occupational medicine clinics. The 95 % confidence intervals of the difference between corporate physical therapy clinics and other clinical settings for doropathy ranged from 2.3 to 21.7 fewer units for hospital-based clinics to 18.6–38.8 fewer units for occupational medicine clinics, while the 95 %-confidence intervals of the difference between corporate physical therapy clinics and other clinical settings for sprains or strains ranged from 7.7 to 12.5 fewer units for private physical therapy clinics to 19.3–27.0 fewer units for occupational medicine clinics.

Corporate physical therapy clinics and hospital-based clinics had higher means for the number of visits for patient conditions classified as fracture or dislocation but were not different from one another (Table 6). There were no significant differences in the number of units between these settings for patients classified as other.

#### Proportion of Units of Physical Agents to Total Units

Occupational medicine clinics and physician offices had a significantly higher proportion of units of physical agents/total units over the episode of care for subjects’ conditions classified as arthropathy, dorsopathy, or sprains or strains. Occupational medicine clinics had a significantly higher proportion of units of physical agents/total units over the episode of care for subjects classified as fractures or dislocations when compared to the other 4 clinical settings. The 95 %-confidence intervals of the differences between occupational medicine clinics and other clinical settings ranged from 0.07 to 0.18 less for physician offices to 0.13–0.24 less for corporate physical therapy clinics. Occupational medicine clinics and physician offices had a significantly higher proportion of units of physical agents/total units over the episode of care for subjects’ conditions classified as arthropathy, dorsopathy, or sprains strains. There were no significant differences in the proportion of units of physical agents/total units over the episode of care among groups for patients classified as other.

Corporate physical therapy clinics and private physical therapy practices had a significantly lower proportion of units of physical agents/total units over the episode of care for subject conditions classified as arthropathy. Corporate physical therapy clinics had a significantly lower proportion of units of physical agents/total units over the episode of care for subject conditions classified as dorsopathy, sprains or strains, or fractures or dislocations compared to the other practice settings (Table 6).

### Differences in Billing Between Practice Setting Adjusted for Body-part Treated Surgically

#### Number of Visits per Episode

All comparisons were significant at the *p* < .001 level. Corporate physical therapy clinics had the highest mean number of visits during the episode of care for subjects classified as receiving rehabilitation associated with surgical treatment to the upper extremity, back, or multiple areas, compared to the other practice settings. Hospital-based out-patient physical therapy clinics had the highest mean number of visits during the episode of care for subjects classified as receiving rehabilitation associated with surgical treatment to the lower extremity, neck, or hand compared to the other practice settings (Table 7).

**Table 7:** The mean (standard deviation) for the primary measures by practice setting and body region that was treated surgically

Practice setting	Body region (s) treated	Number of cases	Mean visits per episode	SD	Mean units per episode	SD	Ratio passive/total units	SD
Physician offices	Upper extremity	219	18.12	14.02	75.66	67.81	.272	.21
	Lower extremity	90	19.14	18.13	90.93	107.86	.262	.23
	Back	77	12.12	9.73	43.70	46.53	.307	.22
	Neck	48	15.87	15.21	59.91	60.95	.291	.22
	Hand	17	16.71	12.93	84.47	77.76	.455	.18
	Multiple	570	18.41	16.56	77.46	86.79	.262	.20
	Total	1,021	17.79	15.72	75.00	82.09	.272	.21
Corporate physical therapy clinics	Upper extremity	1,066	20.90	17.66	113.15	115.82	.194	.15
	Lower extremity	497	20.97	17.30	123.05	193.68	.152	.15
	Back	316	17.17	13.98	88.64	84.38	.166	.18
	Neck	172	17.83	16.24	91.79	94.97	.181	.15
	Hand	55	19.22	12.66	87.32	70.54	.238	.13
	Multiple	3,163	21.86	17.11	117.46	118.41	.191	.15
	Total	5,269	21.14	17.05	114.23	124.52	.187	.15
Occupational medicine	Upper extremity	28	12.18	12.42	54.17	59.23	.381	.16
	Lower extremity	18	16.78	15.00	70.72	64.44	.401	.20
	Back	6	13.17	4.57	47.00	14.54	.438	.07
	Neck	4	12.75	8.01	51.50	31.16	.480	.12
	Hand	8	9.75	7.88	49.00	45.81	.281	.28
	Multiple	78	13.24	10.24	57.52	51.96	.351	.20
	Total	142	13.27	11.03	57.44	53.04	.367	.20
Hospital-based	Upper extremity	73	16.19	13.15	65.63	62.77	.191	.19
	Lower extremity	27	22.63	18.28	106.59	100.07	.127	.18
	Back	19	14.37	12.62	54.63	51.14	.218	.21
	Neck	9	23.00	18.31	127.77	136.35	.248	.18
	Hand	8	24.00	20.73	103.62	90.07	.293	.16
	Multiple	250	18.04	14.72	78.49	85.82	.197	.18
	Total	386	18.07	14.88	78.52	83.77	.195	.18
Private physical therapy practices	Upper extremity	1,912	18.46	15.50	80.93	85.90	.233	.19
	Lower extremity	789	18.94	14.77	80.15	75.26	.198	.18
	Back	570	16.19	13.36	66.75	68.91	.196	.19
	Neck	264	14.92	13.55	60.60	66.25	.232	.21
	Hand	84	17.30	14.92	74.17	91.75	.286	.20
	Multiple	5,942	19.30	15.28	83.42	84.81	.223	.19
	Total	9,561	18.78	15.15	80.95	83.16	.222	.19
Entire sample	Upper extremity	3,298	19.12	16.12	90.42	96.37	.223	.18
	Lower extremity	1,421	19.71	16.01	96.22	132.72	.187	.18
	Back	988	16.13	13.31	71.60	73.50	.197	.19
	Neck	497	16.15	14.79	72.47	79.83	.222	.20
	Hand	172	17.81	14.15	79.59	82.15	.287	.19
	Multiple	10,003	19.98	15.97	93.52	98.03	.216	.18
	Total	16,379	19.41	15.84	91.03	99.48	.215	.18

#### Number of Units per Episode

All comparisons were significant at the *p* < .001 level. Corporate physical therapy clinics had the highest mean number of units during the episode of care for subjects classified as receiving rehabilitation associated with surgical treatment to the upper extremity, lower extremity, back, or multiple areas compared to the other practice settings. Hospital-based practices had the highest mean number of units during the episode of care for subjects classified as receiving rehabilitation associated with surgical treatment to the neck, or hand compared to the other practice settings (Table 7).

#### Proportion of Units of Physical Agents to Total Units

All comparisons were significant at the *p* < .001 level. Occupational medicine clinics and physician offices had a significantly higher proportion of units of physical agents/total units over the episode of care for subjects’ classified as receiving rehabilitation associated with surgical treatment to each of the body-parts, when compared to the other practice settings. Corporate physical therapy clinics and hospital-based out-patient physical therapy clinics had a significantly lower proportion of units of physical agents/total units over the episode of care for subjects’ classified as receiving rehabilitation associated with surgical treatment to multiple body parts, compared to the other practice settings. Corporate physical therapy clinics had a significantly lower proportion of units of physical agents/total units over the episode of care for all other body parts classified as receiving rehabilitation associated with surgical treatment, compared to the other practice settings (Table 7).

### Differences in Billing Between Practice Setting Adjusted for Body-part Treated Non-Surgically

#### Number of Visits per Episode

All comparisons were significant at the *p* < .001 level. Corporate physical therapy clinics had the highest mean number of visits during the episode of care for subjects classified as receiving rehabilitation associated with non-surgical treatment for each of the body-parts treated, compared to the other practice settings. Occupational medicine had the lowest mean number of visits for each of these conditions, compared to the other practice settings (Table 8).

**Table 8:** The mean (standard deviation) for the primary measures by practice setting and body region that was treated non-surgically

Practice setting	Body region (s) treated	Number of cases	Mean visits per episode	SD	Mean units per episode	SD	Ratio passive/total units	SD
Physician offices	Upper extremity			10.31	37.41	49.10	.380	.232
	Lower extremity	488	8.47	8.90	33.61	43.29	.352	.253
	Back	1,566	8.29	8.94	34.00	48.34	.385	.257
	Neck	193	10.22	9.70	39.39	41.53	.338	.270
	Hand	171	8.17	10.16	34.15	52.89	.437	.269
	Multiple	1,206	8.68	8.22	34.17	40.53	.344	.251
	Total	4,078	8.64	9.00	34.64	45.54	.368	.254
Corporate physical therapy clinics	Upper extremity	1,688	11.71	10.98	57.57	64.59	.229	.179
	Lower extremity	1,737	10.39	9.40	52.28	59.55	.207	.178
	Back	5,784	9.81	9.13	47.61	54.67	.232	.189
	Neck	568	11.83	9.92	56.90	64.28	.226	.195
	Hand	657	9.00	8.00	42.71	49.24	.311	.177
	Multiple	5,916	10.85	9.83	53.79	66.48	.248	.193
	Total	16,350	10.49	9.60	51.50	60.98	.238	.189
Occupational medicine	Upper extremity	394	7.16	8.64	32.84	39.82	.405	.219
	Lower extremity	412	6.46	5.98	27.94	27.90	.356	.244
	Back	1,278	6.11	6.39	27.65	30.17	.409	.220
	Neck	54	11.17	16.01	39.50	51.52	.372	.235
	Hand	281	5.28	4.40	25.64	28.71	.428	.233
	Multiple	967	6.85	9.16	31.24	41.95	.431	.215
	Total	3,386	6.50	7.66	29.34	35.22	.410	.224
Hospital-based	Upper extremity	241	10.29	11.55	44.34	61.87	.316	.224
	Lower extremity	195	8.43	8.59	34.11	41.27	.323	.242
	Back	720	8.04	9.73	35.08	82.36	.335	.243
	Neck	36	10.81	9.62	39.66	36.20	.300	.281
	Hand	87	6.78	5.48	29.32	28.73	.443	.224
	Multiple	660	8.77	8.89	35.94	40.70	.317	.251
	Total	1,939	8.60	9.47	36.25	61.61	.329	.244
Private physical therapy practices	Upper extremity	3,111	10.78	11.77	46.56	59.50	.295	.217
	Lower extremity	2,667	9.67	8.86	40.38	46.19	.281	.223
	Back	10,082	9.39	9.35	38.71	44.89	.312	.234
	Neck	1,066	11.64	12.39	47.64	60.73	.303	.260
	Hand	917	8.29	8.64	33.39	39.32	.361	.240
	Multiple	10,331	10.27	9.94	42.63	50.28	.300	.226
	Total	28,174	9.94	9.94	41.34	49.40	.304	.230
Entire sample	Upper extremity	5,888	10.68	11.29	48.00	59.71	.291	.214
	Lower extremity	5,499	9.51	8.91	42.38	49.96	.271	.221
	Back	19,430	9.16	9.15	40.12	49.66	.301	.229
	Neck	1,917	11.52	11.53	49.17	59.80	.286	.248
	Hand	2,113	8.04	8.11	35.15	42.71	.364	.228
	Multiple	19,080	10.12	9.78	44.75	55.10	.294	.223
	Total	53,927	9.74	9.68	42.98	53.06	.296	.226

#### Number of Units per Episode

All comparisons were significant at the *p* < .001 level. Corporate physical therapy clinics had the highest mean number of units during the episode of care for subjects classified as receiving rehabilitation associated with non-surgical treatment for each of body-parts treated, compared to the other practice settings. Occupational medicine had the lowest mean number of visits for each of these conditions, compared to the other practice settings (Table 8).

#### Proportion of Units of Physical Agents to Total Units

All comparisons were significant at the *p* < .001 level. Occupational medicine clinics and physician offices had a significantly higher proportion of units of physical agents/total units over the episode of care for subjects’ classified as receiving rehabilitation associated with non-surgical treatment for the lower extremity, compared to the other practice settings. Occupational medicine clinics had a significantly higher proportion of units of physical agents/total units over the episode of care for subjects’ classified as receiving rehabilitation associated with non-surgical treatment for the upper extremity, back, neck and multiple sites, compared to the other practice settings. Hospital-based out-patient physical therapy clinics had significantly higher proportion of units of physical agents/total units over the episode of care for subjects’ classified as receiving rehabilitation associated with non-surgical treatment of the hand, compared to the other practice settings. Corporate physical therapy clinics had a significantly lower proportion of units of physical agents/total units over the episode of care for subject conditions classified as receiving rehabilitation associated with non-surgical treatment for each of body-parts treated receiving care, compared to the other practice settings (Table 8).

### Differences in Billing Between Practice Setting Based Upon Geographic Location

Patients receiving care in facilities in the mid-Atlantic and East North Central regions of the United States received significantly more visits (95 % CI = 1.67 to 7.40, *p* < .001) and units (95 % CI = 7.6–46.2, *p* < .001) during the episode of care compared to other regions (Table 9). When data were adjusted for practice setting, diagnosis, body-part treated or surgical status the significance and directionality of differences were not changed.

**Table 9:** The mean (standard deviation) for the primary measures listed for practice setting and the geographic region where care was provided

Practice setting	Geographic region	Number of cases	Visits per episode	SD	Units per episode	SD	Ratio passive/total units	SD
Physician offices	New England	390	11.05	9.07	37.65	39.17	.277	.240
	Middle Atlantic	945	15.13	16.65	65.93	83.19	.330	.239
	South Atlantic	1,346	9.89	9.05	38.74	42.99	.242	.228
	East North Central	47	13.47	9.84	68.02	67.53	.276	.250
	East South Central	203	8.96	8.35	32.06	33.64	.235	.205
	West North Central	20	9.75	8.77	45.65	51.15	.259	.230
	West South Central	140	9.73	9.78	44.68	48.61	.139	.227
	Mountain	31	13.29	11.40	60.90	65.42	.375	.282
	Pacific	1,977	8.63	9.55	35.39	52.23	.475	.214
	Total	5,099	10.47	11.29	42.73	57.17	.349	.249
Corporate physical therapy clinics	New England	1,033	12.37	11.48	57.03	69.06	.312	.193
	Middle Atlantic	6,469	15.39	15.53	85.86	114.56	.243	.185
	South Atlantic	6,879	11.34	9.91	53.66	59.42	.184	.167
	East North Central	2,426	16.21	14.66	88.22	91.27	.204	.163
	East South Central	513	10.64	9.68	52.20	62.85	.204	.179
	West North Central	1,499	11.30	10.98	50.81	61.89	.223	.202
	West South Central	642	10.81	10.07	56.26	62.67	.182	.184
	Mountain	820	13.69	11.73	73.57	72.61	.268	.166
	Pacific	1,337	9.38	8.29	35.11	43.91	.320	.186
	Total	21,618	13.08	12.73	66.79	85.54	.225	.183
Occupational medicine	New England	237	5.19	2.97	20.45	13.03	.590	.150
	Middle Atlantic	347	12.83	18.76	52.44	73.58	.478	.161
	South Atlantic	1,384	6.24	5.85	26.40	27.53	.254	.211
	East North Central	2	3.00	1.41	15.50	10.60	.744	.007
	East South Central	121	6.55	4.68	43.32	36.53	.484	.208
	West North Central	28	8.57	6.65	28.53	26.63	.268	.243
	West South Central	28	6.46	4.55	36.50	35.18	.188	.192
	Mountain	25	9.16	8.69	34.20	32.77	.264	.276
	Pacific	1,356	5.99	4.69	29.47	30.56	.518	.143
	Total	3,528	6.77	7.93	30.47	36.52	.408	.223
Hospital-based	New England	485	6.90	6.44	31.09	30.41	.466	.205
	Middle Atlantic	545	13.73	14.21	61.56	90.30	.238	.220
	South Atlantic	504	10.89	11.37	44.21	63.31	.281	.234
	East North Central	115	9.79	8.39	30.93	30.14	.233	.254
	East South Central	396	9.49	8.97	41.15	47.75	.230	.220
	West North Central	54	7.50	6.78	24.98	30.60	.287	.310
	West South Central	39	8.05	6.64	33.20	26.96	.203	.223
	Mountain	38	7.84	6.23	27.00	23.01	.325	.237
	Pacific	149	9.64	15.18	41.41	125.37	.419	.180
	Total	2,325	10.17	11.13	43.27	67.64	.307	.241
Private physical therapy practices	New England	1,918	13.94	13.58	59.53	80.45	.318	.236
	Middle Atlantic	7,798	15.74	16.76	69.11	83.57	.334	.230
	South Atlantic	12,649	11.59	10.21	48.22	50.86	.224	.208
	East North Central	1,986	13.08	12.00	57.24	67.79	.253	.220
	East South Central	2,253	10.42	9.56	45.48	51.65	.263	.224
	West North Central	1,558	12.37	11.95	51.12	57.99	.262	.220
	West South Central	1,249	10.99	10.13	49.64	55.42	.228	.219
	Mountain	2,515	11.43	10.79	55.49	64.10	.316	.205
	Pacific	5,809	9.03	7.89	30.68	33.60	.354	.214
	Total	37,735	12.18	12.11	51.37	62.22	.283	.224
Total sample	New England	4,063	11.91	11.90	51.12	68.52	.346	.232
	Middle Atlantic	16,104	15.43	16.24	75.04	97.66	.297	.218
	South Atlantic	22,762	11.07	9.96	47.89	52.84	.216	.200
	East North Central	4,576	14.66	13.50	73.10	82.19	.227	.195
	East South Central	3,486	10.13	9.35	45.12	51.90	.257	.221
	West North Central	3,159	11.73	11.40	50.29	59.40	.244	.215
	West South Central	2,098	10.73	10.00	50.85	56.87	.207	.210
	Mountain	3,429	11.93	11.02	59.39	66.32	.305	.199
	Pacific	10,628	8.62	8.17	32.11	41.36	.394	.214
	Total	70,305	12.00	12.13	54.17	69.84	.277	.219

States included in each region are as follows: New England (CT, ME, MA, NH), Middle Atlantic (NJ, NY, PA), East North Central (IL, IN, MI, OH, WI), West North Central (IA, KS, MN, MO, NE, ND, SD), South Atlantic (DE, DC, FL, GA, MD, NC, SC, VA, WV), East South Central (AL, KY, MS, TN), West South Central (AR, LA, OK, TX), Mountain (AZ, CO, ID, MT, NV, NM, UT, WY), Pacific (AL, CA, HA, OR, WA)

Patients receiving care in facilities in the Pacific and New England Regions received a higher proportion of physical agents to total units (95 % CI .02–.21) during their episode of care compared to all other geographic areas. Patients receiving care in facilities in the West South Central, South Atlantic and East regions received a lower proportion of physical agents to total units (95 % CI .08–.17) during the episode of care compared to all other geographic areas. The significance and directionality of differences was not changed when data were adjusted for practice setting, diagnosis, body-part treated or surgical status.

## Discussion

### Main Findings

Our goal was to determine if the utilization and type of physical rehabilitation care for injured workers differed based upon the setting in which the care was provided. In the present study, numerous significance differences were identified. Utilization of physical rehabilitation treatment was significantly different among settings regardless of ICD-9-CM classification, body-part treated, surgical or non-surgical intervention, and geographic area in which treatment was provided. Patients receiving care in corporate physical therapy clinics and private physical therapy practices consistently had more visits and overall units of treatment during their episode of care than did the other practice settings addressed in this study. The exact reasons for this observation are unknown. One possible explanation is that these facilities may have typically treated patients who required more care, i.e., those with more complex and prognostically unfavorable conditions than those seen in other settings. In our sample, corporate physical therapy clinics and private physical therapy practices treated higher than expected frequencies of patients who had surgical intervention, and would likely require substantial care, compared to other settings. However, these subjects only accounted for 24.1 % of the total number of subjects treated by corporate physical therapy clinics and 25.3 % of the total number of subjects treated by private physical therapy practices. There were no meaningful between-group changes in our findings after the analysis was adjusted for surgical intervention. The remainder of between-setting frequencies of potential predictor variables (Table 2) recorded at the inception of care did not reflect meaningful differences between corporate physical therapy clinics and private physical therapy practices compared to other settings. It is not known if other patient-specific characteristics such as job description and the presence of bio-behavioral factors or other co-morbidities, that were not addressed in this study could explain the between-setting differences in physical rehabilitation utilization.

A second possible explanation for the higher number of visits and units utilized by corporate physical therapy clinics and private physical therapy practices is that more total treatment provides a more effective outcome than less overall treatment. This contention could be addressed by a comparison of outcome measures reflecting important status changes such as functional recovery and/or return to work. Unfortunately, these data were not available; therefore, no judgments may be made on the overall “value” of care between settings.

The treatment emphasis was also significantly different between settings. Occupational medicine clinics and physician offices had higher proportions of physical agents to total units than did other settings. This finding remained consistent after the analyses were adjusted for body-part treated, surgical or non-surgical intervention and geographic area in which treatment was provided. The reason for this difference is unknown. Although the body of supporting evidence is limited, physical agents have been primarily advocated as a means to control pain in people with acute injuries [22]. The majority of patients seen in occupational medicine clinics (68.2 %) received care within 30 days of injury, which may explain the higher usage of physical agents in this setting. However, physician offices had a predominance of patients with more chronic conditions, i.e., greater than 90 days from injury to start of care (Table 2), and these settings had significantly higher proportions of physical agents to total units compared to corporate physical therapy clinics and private physical therapy practices. Another argument for the high usage of physical agents in occupational medicine clinics and physician offices is that these settings may use more non-physical therapist “care-extenders” to provide treatment than do corporate physical therapy clinics or physical therapy private practices [14]. We are unable to address the issue from our dataset. Further study is needed to determine the relationship between the specific person delivering care and the type of treatment delivered.

Corporate physical therapy clinics and private physical therapy practices had significantly lower proportions of physical agents to total units compared to other settings, indicating a higher usage of therapeutic procedures that are supported by evidence-based treatment guidelines [24–34]. This finding is important because recent evidence has suggested that the early and sustained involvement of injured workers in the active process of their care, i.e., performing exercises and activities that encourage patients to move injured body-parts, may have both physiological and psychological benefits that exceed those provided by physical agents [35].

An unexpected finding was the large difference in treatment utilization between geographic regions regardless of practice setting, diagnosis, body-part treated or surgical intervention. The reason for this finding is unknown, but may reflect variations in local reimbursement policies.

### Practical Implications and Further Research

The implications of our findings are that, regardless of ICD-9-CM code classification, body-part treated, and the presence of surgical or non-surgical intervention, there are likely to be significant differences in physical rehabilitation utilization and treatment emphasis for injured workers between practice settings. Patients treated in corporate physical therapy clinics and private physical therapy practices are likely to receive more care than those treated in occupational medicine clinics, physician offices or hospital-based outpatient clinics. Physical rehabilitation care provided in corporate physical therapy clinics and private physical therapy practices is likely to have the greatest emphasis on exercise- and manual therapy-based treatments, while care provided in occupational medicine clinics and physician offices will have a greater emphasis on the use of physical agents. These findings, although preliminary, suggest the need for stakeholders to further investigate the role of practice setting on overall cost-effectiveness of physical rehabilitation provided to injured workers [2–4, 19, 36–38].

### Strengths and Limitations

This study examined a large dataset representing urban, suburban and rural physical rehabilitation delivery to injured workers throughout the United States. The analysis was adequately powered to detect between-setting differences; however, there was an imbalance in frequency of subjects from different clinical settings. The majority (84.4 %) of the subjects received care from private physical therapy practices and corporate physical therapy clinics. Although definitive data are missing, we believe that this distribution of care is likely to be similar to actual clinical practice. The age, gender mix and other demographic characteristics of our sample are similar to other studies assessing care for injured workers; however, our findings can only be generalized to the population of people receiving physical rehabilitation for a musculoskeletal problem associated with a workers’ compensation claim.

## Conclusions

There were significant differences in billing for physical rehabilitation services between practice settings for patients receiving workers’ compensation. Corporate and private physical therapy practices billed for more total visits and total units over the episode of care than did other practice settings. Corporate physical therapy clinics billed for a higher proportion of those interventions supported by evidence-based guidelines (exercise and manual therapy) than did other practice settings. Occupational medicine clinics and physician offices billed for a higher proportion of those interventions generally not supported by evidence-based guidelines (physical agents) over the course of care than did other clinics.
